# Cardioprotection Effects of Sevoflurane by Regulating the Pathway of Neuroactive Ligand-Receptor Interaction in Patients Undergoing Coronary Artery Bypass Graft Surgery

**DOI:** 10.1155/2017/3618213

**Published:** 2017-02-28

**Authors:** Jinquan Wang, Jian Cheng, Chao Zhang, Xiaojun Li

**Affiliations:** ^1^Department of Anesthesiology, Southwest Hospital, Chongqing 400038, China; ^2^Department of Anesthesiology, The People's Hospital of Bishan County, Chongqing 402760, China

## Abstract

This study was designed to identify attractor modules and further reveal the potential biological processes involving in sevoflurane-induced anesthesia in patients treated with coronary artery bypass graft (CABG) surgery. Microarray profile data (ID: E-GEOD-4386) on atrial samples obtained from patients receiving anesthetic gas sevoflurane prior to and following CABG procedure were downloaded from EMBL-EBI database for further analysis. Protein-protein interaction (PPI) networks of baseline and sevoflurane groups were inferred and reweighted according to Spearman correlation coefficient (SCC), followed by systematic modules inference using clique-merging approach. Subsequently,* attract *method was utilized to explore attractor modules. Finally, pathway enrichment analyses for genes in the attractor modules were implemented to illuminate the biological processes in sevoflurane group. Using clique-merging approach, 27 and 36 modules were obtained from the PPI networks of baseline and sevoflurane-treated samples, respectively. By comparing with the baseline condition, 5 module pairs with the same gene composition were identified. Subsequently, 1 out of 5 modules was identified as an attractor based on* attract *method. Additionally, pathway analysis indicated that genes in the attractor module were associated with neuroactive ligand-receptor interaction. Accordingly, sevoflurane might exert important functions in cardioprotection in patients following CABG, partially through regulating the pathway of neuroactive ligand-receptor interaction.

## 1. Introduction

Currently, coronary artery bypass grafting (CABG) surgery has been considered as one of the most effective methods in the treatment of coronary heart disease [1]. However, CABG surgery can cause ischemic injury because of a transient period of local ischemia with temporary occlusion of the target vessel, particularly in patients with poor cardiac contractile function [2]. Various interventions, such as anesthetics, prior to and following myocardial ischemia, have the potential to reduce myocardial ischemic damage to a certain degree [3, 4]. Sevoflurane is a commonly used anesthetic in CABG surgery [5, 6]. Sevoflurane is an inhalation anesthetic that remarkably decreases the size of infarcts as well as Ca^2+^ loading to protect the myocardium against reperfusion injury [7] and it has myocardial protective effect on low risk patients treated with CABG surgery [8]. Moreover, a former study has indicated that sevoflurane reduces the occurrence of late cardiac events in the first year after CABG procedure, which may play roles via downregulating the expression level of platelet endothelial cell adhesion molecule-1 [9]. It is worth noting that several meta-analyses have also confirmed the cardioprotective effects of sevoflurane [10, 11]. Nevertheless, the molecular mechanisms by which sevoflurane exerts its protective effect remain poorly defined.

It is generally known that protein interactions exert significant functions in the cellular processes. With the development of high-throughput technologies, it has been found that protein-protein interactions (PPIs) can be used to study proteins systematically and to prioritize disease-related genes and pathways [12, 13]. However, the high false positive and false negative rates in PPI might influence the performance of some discovery algorithms. Thus, many computational methods have been created to evaluate the reliability of PPIs. For example, Chua et al. [14] have indicated that utilization of Functional Similarity Weight (FS-Weight) can eliminate unreliable interactions and add new ones with high FS-Weight scores. Moreover, an iterative scoring method has been applied to predict new interactions and evaluate the reliability of PPIs, and this method exhibited better performance than FS-Weight [15]. In addition, Liu and colleagues have developed an algorithm called clustering-based on maximal cliques to extract complexes from the weighted PPI networks [16]. It is crucial to effectively integrate multiomics data into such an analysis. For example, a former study integrated PPI with microarray data to construct tissue-specific PPI networks for 60 tissues and used them to prioritize disease-related genes [17]. Chu and Chen [18] constructed a cervical carcinoma-disturbed PPI network by combining PPI and gene expression profile to extract gain- and loss-of-function genes which might be potential drug targets. However, it is challenging to study multiple diseases synchronously. Thus, it is crucial to study the behavior of modules across specific conditions in a controlled manner to understand the modus operandi of disease mechanisms and to implicate novel genes and then to develop effective treatment methods [19]. Although several significant genes showing no difference might not be detected via their own behavior, they can be identified when these genes worked together with other genes (e.g., as modules). Accordingly, several studies have identified functional modules from PPI networks [20, 21].

In our study, in order to determine the influence of sevoflurane on postoperative recovery in patients following CABG, module analysis based on the reweighted PPI networks was used to indicate the potential mechanisms of sevoflurane effect and to identify the underlying biosignatures. In brief, gene expression profile E-GEOD-4386 was recruited from the EMBL-EBI database. Then, the PPI networks of baseline and sevoflurane groups were constructed and reweighted on the basis of Spearman correlation coefficient (SCC), following by module identification from the PPI networks using clique-merging algorithm. Afterwards,* attract* method was utilized to select attractor modules. Finally, pathway enrichment analyses for genes in attractor modules were implemented to illuminate the biological processes in sevoflurane group.

## 2. Materials and Methods

### 2.1. Acquisition of Gene Expression Profile

The gene expression profile under the series number of E-GEOD-4386 [22] was recruited from the EMBL-EBI database based on the platform of A-AFFY-44, Affymetrix GeneChip Human Genome U133 Plus 2.0 [HG-U133_Plus_2]. This profile E-GEOD-4386 contained 40 samples including data from patients undergoing CABG procedure with propofol treatment (*n* = 10), sevoflurane treatment (*n* = 10), and control samples (*n* = 20). The control samples were obtained from patients before CABG procedure. In the current study, in order to determine the influence of sevoflurane on postoperative recovery in patients undergoing CABG, we selected 10 atrial samples obtained from patients undergoing CABG surgery with sevoflurane treatment (sevoflurane group). Moreover, 10 control samples obtained from the same patients prior to CABG surgery were selected (baseline group).

All the patients had three-vessel coronary artery disease, and the average age of patients was 65.2 years (range 50–80 years). Patients with hemodynamic instability were not included. Furthermore, patients were treated preoperatively with beta-blocker (*n* = 9), Ca^2+^ blocker (*n* = 5), nitrates (*n* = 5), and statins (*n* = 5), respectively.

None of the patients had postoperative myocardial infarction, renal damage, or cerebrovascular injury. No postoperative mechanical or medical inotropic support was required.

More information about the patient characteristics were shown in the study of Srihari and Ragan (see [19]).

### 2.2. Pretreatment of Raw Data

Original gene expression data were pretreated by conducting background correction using robust multiarray average (RMA), quantile normalization, perfect match (PM)/mismatch (MM) correction via MicroArray Suite (MAS), and Media Polish Summarization of the expression measures. Next, we transformed probe IDs into gene symbols.

### 2.3. Inferring PPI Networks of Baseline and Sevoflurane Groups

Because complicated cellular processes are usually regulated by tightly connected proteins and interactions with weight scores reflect the reliability of interactions, the PPI networks were further analyzed on the basis of the public database STRING (Search Tool for the Retrieval of Interacting Genes/Proteins, version 9.1, http://string-db.org/) [23]. First, all human PPIs were obtained from the STRING database. Then, proteins without expression value were eliminated and the repeated IDs for a given gene were reduced to a single one. To minimize false positive rate, protein interactions with combine-score ≥ 0.8 were remained, which included 8008 nodes and 48,930 interactions. By intersecting with the gene expression data, a new PPI network with 7967 nodes and 48,930 interactions was constructed.

The interactions in the networks of baseline and sevoflurane groups were reweighted using SCC. As documented, SCC is used to evaluate the strength of association of two coexpressed variables and the value ranges from −1 to 1 inclusive [24]. The weight value of a pair of proteins was determined as the absolute value of SCC of the corresponding gene interaction. If SCC value is positive, there is a positive linear correlation between the two proteins. We only chose the interactions with significant SCC values (*P* < 0.05) to construct the conditional-specific PPI networks of baseline and sevoflurane groups.

### 2.4. Identification of Modules from Conditional-Specific PPI Networks

In our analysis, we identified the conditional-specific modules using modules-identification algorithm in Genelibs (http://www.genelibs.com/gb/) based on clique-merging method [25].

This algorithm included two steps:

1. All maximal cliques were found from the reweighted PPI networks and sorted in order according to their weighted interaction density (WID).
2. Highly overlapped cliques were merged.

Cliques algorithm introduced by Tomia and colleagues applied a depth-first search method to enumerate all maximal cliques. The score of clique *C* was determined as its WID and was computed according to the formula:

(1) $$\begin{array}{c} \text{score}\left(\;C\;\right)=\frac{\sum_{u\in C,v\in C}w\left(u,v\right)}{\left|C\right|\left(\left|C\right|-1\right)}. \end{array}$$

In this formula, score(*C*) was the WID of clique *C*; and *w*(*u*, *v*) was on behalf of the weight of the interaction between *u* and* v*.

Many maximal cliques might overlap with each other, in order to lower the result size, the highly overlapped maximal cliques were removed or merged. We computed weighted interconnectivity (WIC) between 2 cliques to determine whether these 2 overlapped cliques were merged or not.

(2) scoreC1,C2=∑u∈C1−C2∑v∈C2wu,vC1−C2·C2·∑u∈C2−C1∑v∈C1wu,vC2−C1·C1

In this formula, score(*C*_1_, *C*_2_) was the WIC between proteins of *C*_1_,  *C*_2_.

The cliques were ranked in descending sequence of their WIC, named as clique [*C*_1_], [*C*_2_], [*C*_3_]… [26]. In brief, for every maximal clique [*C*_*i*_], we repeatedly checked whether clique [*C*_*j*_] existed. If the ratio of the overlap between [*C*_*i*_] and [*C*_*j*_] was greater than *t*_0_ which was an overlap-threshold, and if there was such a clique [*C*_*j*_], the WIC value of [*C*_*i*_] and [*C*_*j*_] was calculated. When WIC was higher than *t*_*m*_, a predefined merge-threshold, [*C*_*j*_] was merged into [*C*_*i*_] to develop a module. Otherwise, maximal clique [*C*_*j*_] was removed. Herein, *t*_0_ = 0.5 and *t*_*m*_ = 0.25 were considered as the threshold.

### 2.5. Comparison of Modules between Baseline and Sevoflurane-Treated Conditions

In the current study, we used systematic method to study the behavior of modules between baseline and sevoflurane-treated conditions in a controlled manner to understand the modus operandi of disease to implicate novel genes [19]. *H*_*S*_ and *H*_*B*_ were on behalf of the PPI networks of sevoflurane-treated and baseline samples, respectively. The module sets *S* = {*S*_1_, *S*_2_,…, *S*_*k*_} and *M* = {*M*_1_, *M*_2_, …, *M*_*m*_} were, respectively, selected from *H*_*S*_ and *H*_*B*_. For module *S*, the module correlation density (MCD) was calculated as

(3) $$\begin{array}{c} d_{CC}\left(\;S_{i}\;\right)=\frac{\sum_{u,v\in S_{i}}SCC\left(\left(u,v\right),N\right)}{\left|S_{i}\right|\left(\left|S_{i}\right|-1\right)}. \end{array}$$

Similarly, we calculated the MCDs of modules in the baseline condition. Then, we utilized Jaccard similarity to extract the module pairs having either the similar or same gene composition. In our study, Jaccard score was set as 0.7.

### 2.6. Identification of Attractor Modules Using Attract Method

We employed these module pairs to identify the attractor modules using* attract *method [27]. Based on ANOVA model, Fisher's test was implemented for genes in attractors and the *F*-statistic value for gene *a* was calculated as follows:

(4) $$\begin{array}{c} F^{\left(a\right)}=\frac{\left(1/\left(K-1\right)\right)\sum_{k=1}^{K}r_{k}{\left[y_{\cdot k}^{\left(a\right)}-y_{\cdot \cdot }^{\left(a\right)}\right]}^{2}}{\left(1/\left(N-K\right)\right)\sum_{k=1}^{K}\sum_{b=1}^{rb}{\left[y_{bk}^{\left(a\right)}-y_{\cdot \cdot }^{\left(a\right)}\right]}^{2}}, \end{array}$$

where *N* meant the total number of samples; *r*_*k*_ was on behalf of each cell type *k* = 1, …, *K*;  *y* stood for the mixed effect model; *b* stood for the corresponding expression value in each replicate sample. Afterwards, *T*-test was used to examine the *F*-statistics values, and the *P* values were obtained. Then, the *P* values were adjusted based on false discovery rate (FDR) using Benjamini-Hochberg method [28]. Remarkably, the modules with FDR < 0.05 were considered as the attractor modules.

### 2.7. Pathway Enrichment Analysis

Frequently, the development of diseases is caused by the alteration of pathways participated in the biological process. For this reason, pathways enrichment analysis for attractor module genes was carried out. In our study, all reference pathways were downloaded from Kyoto Encyclopedia of Genes and Genomes (KEGG) database. Subsequently, genes in attractor modules were aligned to the reference pathways to extract the significant pathways. Significant pathways were selected based on the FDR < 0.01.

### 2.8. Statistical Analysis

The fold change and Feature Extraction software 10.7 were applied to analyze the statistical significance of the microarray results. The raw data were normalized using the Quantile Algorithm (Agilent Technologies). The FDR was computed to adjust the original *P* values. The threshold value utilized to designate attractor module was FDR < 0.05, and the cut-off criteria for significant pathways was FDR < 0.01. *P* < 0.05 was considered to indicate a statistically significant difference. Fisher's test was employed to identify the attractor modules. In our study, we used SPSS version 18.0 (SPSS Inc., Chicago, IL, USA) for statistical analysis.

## 3. Results

### 3.1. Disruptions in PPI Networks

**Figure 1:**
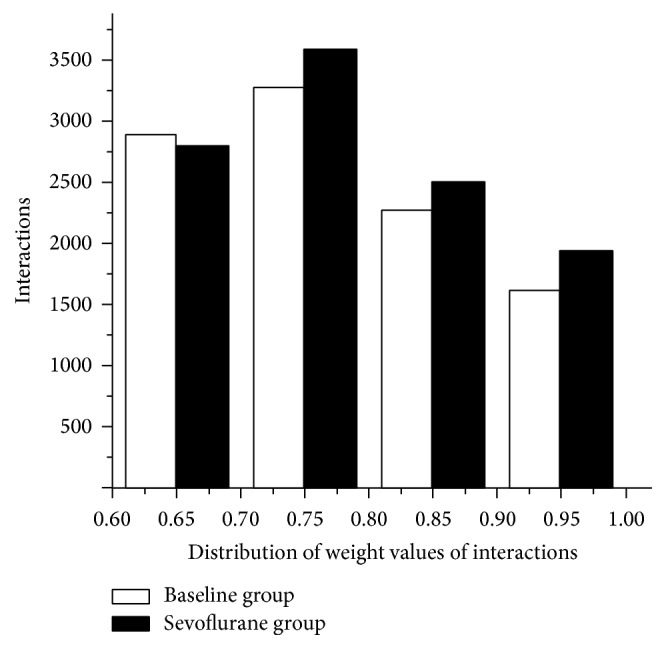
Weight values distribution of interactions in baseline and sevoflurane protein-protein interaction (PPI) networks.

Through the mapping between each probe and the corresponding official symbol, we obtained one expression profile data containing 20,389 genes. Next, we investigated the interactions with significant SCC values and obtained the PPI networks of baseline and sevoflurane groups. The baseline and sevoflurane PPI networks exhibited different number of interactions (10,061 and 10,998 in baseline and sevoflurane group, resp.). The mean weight values of baseline and sevoflurane were 0.778 and 0.783, respectively. From Figure 1, we found that the weight values of the interactions in both baseline and sevoflurane PPI networks ranged from 0.60 to 1.00. Moreover, the number of interactions in sevoflurane PPI network was greater than that in baseline group in the weight value distribution of 0.70–1.00, while the number of interactions of sevoflurane PPI network was smaller in the weight distribution of 0.60–0.70. Kolmogorov-Smirnov test was used to measure the differences of the weight distributions for baseline and sevoflurane. Finally, there were no significant differences in the weight distributions for baseline and sevoflurane groups.

### 3.2. Selection of Attractor Modules

After fast depth-first algorithm, 6461 and 7253 maximal cliques were found in PPI networks of baseline and sevoflurane groups, respectively. Then, we obtained 628 and 729 maximal cliques in baseline and sevoflurane PPI networks after the cliques with nodes less than 5 were removed, and these 628 and 729 maximal cliques were used to perform the module analysis. As listed in Table 1, a total of 27 and 36 modules were identified from the baseline and sevoflurane PPI networks, respectively. Moreover, the average module size of baseline group was slightly greater than that in sevoflurane, and the maximum, minimum, and average WID were approximately the same as those in sevoflurane group. In addition, no difference was observed in maximum, minimum, and average correlations between the two groups of modules (Table 1).

**Figure 2:**
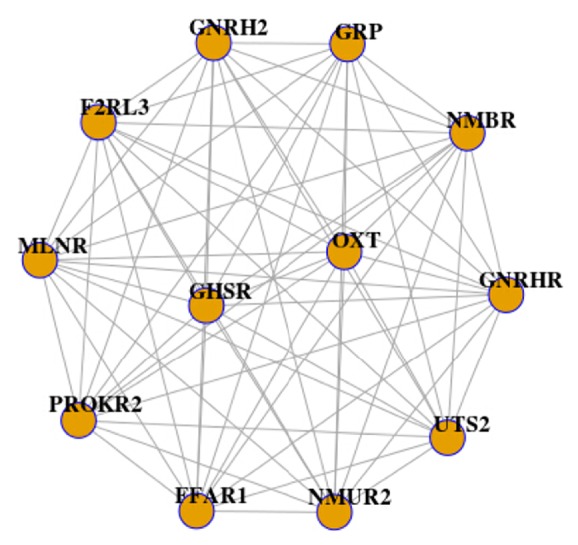
One attractor module involving 12 nodes and 66 interactions. Significantly, weighted interaction density (WID) was significant in this differential module (WID = 0.353 for sevoflurane group and WID = 0.121 for baseline group, *P* < 0.05).

Next, we obtained 5 module pairs with the same composition between two groups according to Jaccard score = 0.7. Subsequently, we utilized* attract *method to identify the attractor modules in these 5 module pairs. Based on the cut-off criteria of FDR < 0.01, only one module including 12 nodes and 66 interactions was differential, as listed in Figure 2. In addition, WID was significant in this differential module (WID = 0.353 for sevoflurane group and WID = 0.121 for baseline group; *P* < 0.05).

### 3.3. Pathway Enrichment Analysis

In order to shed light on relevant cellular processes, pathway-based method was used to analyze the genes in the attractor module. We found that 5 genes (NMUR2, GHSR, NMBR, GNRHR, and F2RL3) in the attractor module were enriched in the pathway of neuroactive ligand-receptor interaction. Then, by looking back and checking the gene expression profile for these genes in our dataset, we would like to know if any of these genes are highly expressed in the disease. Based on |log FC| ≥ 2 and FDR < 0.05, we found that all of these 5 genes were not differentially expressed. Generally, some hidden genes showing no difference by themselves are frequently overlooked, which might be functionally associated with differentially expressed genes. Hence, our result further indicated that this method was available to detect significant modules and several hidden genes exhibiting no difference by themselves yet clustered in a module.

Moreover, we have checked the expression profiles of these genes in other cardio or neuronal diseases and found that these 5 genes were related to other cardio or neuronal diseases. The corresponding result was shown in Table 2 [29–33].

In addition, we have checked whether there were known drugs targeted for genes in the module available from the GeneCards database. We discovered that most genes of this module were the targets of drugs. For example, NMBR gene-targeted drugs included Bombesin and Ranatensin; GNRHR was the target of Degarelix, Nafarelin, Cetrorelix, Goserelin, and Leuprolide; F2RL3 gene was related to Argatroban. Specific information was shown in Table 3.

## 4. Discussion

As documented, anesthetics can regulate gene expression [34] and play important roles in organ protection [35]. Sevoflurane is one of the most frequently used anesthetics in CABG surgery [36]. However, the related mechanisms of the effects of sevoflurane remain unclear. In the current study, in order to determine the protective molecular mechanisms of sevoflurane, the CABG-related dataset E-GEOD-4386 was selected for further analysis. Using clique-merging approach, a total of 27 and 36 modules were obtained from the PPI networks of baseline and sevoflurane-treated samples, respectively. By comparing with baseline condition, there were 5 module pairs with the same gene composition. Significantly, 1 out of 5 modules was an attractor. Moreover, pathway analysis showed that genes in the attractor module were related to neuroactive ligand-receptor interaction.

Neuroactive steroids are hormones that act as regulators of neurotransmitter receptors to either enhance or suppress neuronal activity [37]. The effect of steroid exhibits marked stereoselectivity, suggesting a ligand-receptor interaction. According to the literatures, neuroactive steroid influences the modulation of GABA receptor [38, 39]. More importantly, many anesthetics, including sevoflurane, exert key functions via selectively targeting GABA receptors [40, 41]. A former study has demonstrated that disruption of GABA affects mitochondrial respiration [42]. Another study has suggested that sevoflurane-induced preconditioning mediates cardioprotection via preserving mitochondrial functions during ischemia and perfusion [43]. Based on these, we infer that sevoflurane might serve a crucial role in cardioprotection by influencing the pathway of neuroactive ligand-receptor interaction.

In the functional pathway of neuroactive ligand-receptor interaction, 5 genes NMUR2, GHSR, NMBR, GNRHR, and F2RL3 were involved. Ghrelin, as a peptide hormone, has been identified as an endogenous ligand for the growth hormone secretagogue receptor (GHSR) [44]. Moreover, growing evidence has demonstrated that ghrelin and the receptor GHSR-1a exist in heart, and administration of ghrelin has beneficial cardiovascular effects in animal models [45, 46] and in humans [47]. GNRHR is a receptor located on the surface of pituitary gonadotropin-releasing cells, mammary gland cells, and ovarian cells, and it can bind to gonadotropin-releasing hormone (GnRH). So far, few studies have shown a direct relationship between GNRHR and heart disease. However, a former study has indicated that GnRH agonists for the treatment of prostate cancer increases the risk of heart disease [32]. Based on these, we infer that sevoflurane might exert important roles in cardioprotective effects, at least in part, through targeting GHSR and GNRHR.

However, there were still some disadvantages in our study. We did not collect microarray profiles on different volatile anesthetics for comparative analysis. Hence, our results were restricted to transcriptional changes in the atrial samples induced by sevoflurane anesthesia. Furthermore, the potential biological pathways were only extracted based on bioinformatics analysis rather than well-designed experiments. Therefore, the significant biological pathways and pathway-related genes may require further in-depth verification in animal models. Nevertheless, our study was only designed as a preliminary analysis and our findings might provide theoretical guidelines for similar studies in the future.

## 5. Conclusion

In conclusion, sevoflurane might exert important functions in cardioprotection in patients following CABG, partially through regulating the pathway of neuroactive ligand-receptor interaction.

## Figures and Tables

**Table 1:** Characteristics of modules in baseline and sevoflurane groups.

Module set	Number of modules	Average module size	Correlation		
			Max	Min	Avg
Baseline	27	8.39	0.481115	0.361867	0.423774
Sevoflurane	36	7.53	0.478742	0.345664	0.423940
*P* value			>0.05	>0.05	>0.05

**Table 2:** Pathway- and module-related genes and the evidence of these genes associated with other cardio or neuronal diseases.

Row	Genes	Evidence
1	NMUR2 [26]	[29]
2	GHSR [27]	[30]
3	NMBR [28]	[31]
4	GNRHR [29]	[32]
5	F2RL3 [30]	[33]

**Table 3:** Module-related genes and the drugs targeted for these genes detected from GeneCards database.

Row	Genes	Drugs
1	GHSR	Hexarelin
2	NMBR	Bombesin, Ranatensin
3	GNRHR	Degarelix, Nafarelin, Cetrorelix, Goserelin, Leuprolide
4	F2RL3	Argatroban
5	GRP	Bombesin
6	MLNR	Roxithromycin, Erythromycin, Azithromycin
7	FFAR1	Icosapent, Alpha-linolenic acid
8	OXT	Oxytocin, Carbetocin
